# Hybrid setup for stable magnetic fields enabling robust quantum control

**DOI:** 10.1038/s41598-018-22671-5

**Published:** 2018-03-13

**Authors:** Frederick Hakelberg, Philip Kiefer, Matthias Wittemer, Tobias Schaetz, Ulrich Warring

**Affiliations:** Albert-Ludwigs-Universität Freiburg, Physikalisches Institut, Hermann-Herder-Straße 3, 79104 Freiburg, Germany

## Abstract

Well controlled and highly stable magnetic fields are desired for a wide range of applications in physical research, including quantum metrology, sensing, information processing, and simulation. Here we introduce a low-cost hybrid assembly of rare-earth magnets and magnetic field coils to generate a field strength of $\simeq$10.9 mT with a calculated spatial variation of less than 10^−6^ within a diameter of spherical volume of 150 μm. We characterise its tuneability and stability performance using a single Mg^+^ atom confined in a radio-frequency surface-electrode trap under ultra-high vacuum conditions. The strength of the field can be tuned with a relative precision of ≤2 × 10^−5^ and we find a passive temporal stability of our setup of better than 1.0 × 10^−4^ over the course of one hour. Slow drifts on time scales of a few minutes are actively stabilised by adjusting electric currents in the magnetic field coils. In this way, we observe coherence times of electronic superposition states of greater than six seconds using a first-order field insensitive (clock) transition. In a first application, we demonstrate sensing of magnetic fields with amplitudes of ≥0.2 μT oscillating at $\simeq$2π × 60 MHz. Our approach can be implemented in compact and robust applications with strict power and load requirements.

## Introduction

Quantum technologies^1^ are developed for a wide range of applications in the context of metrology^2^, sensing^3^, information processing^4^, communication^5^, and simulation^6–9^. While different experimental platforms are studied^3,4,6,9,10^, atomic systems, in particular, perform quantum gate operations with highest fidelities^4,11,12^ and present clocks with exceptional precision^13–18^. Generally, advantageous performance of any quantum application in comparison to classical counterparts can be harnessed only when required control fields interplay with a high level of precision, while the system is well isolated from environmental disturbances. For example, static magnetic (quantisation) fields tune and stabilise electronic states of atoms to desired energy splittings which can be addressed by additional control fields for state manipulation. Fidelities of coherent manipulations crucially depend on the performance of any such quantisation fields. For some applications, specific combinations of atomic species and field strengths can be desired and enable the use of so-called first-order field insensitive (clock) transitions^19,20^ that are less sensitive to field fluctuations than others.

The origin of these field insensitive transitions in atomic species with non-zero nuclear magnetic moments lies in variable nuclear and electronic (hyperfine) interaction strengths as a function of an externally applied magnetic field strength^21^. In the low field (Zeeman) regime, electronic and nuclear angular momentum are coupled and the interaction with the magnetic field can be treated as a perturbation. In contrast, in the high field (Paschen-Back) regime, electronic and nuclear angular momentum are decoupled and the hyperfine interaction can be treated as a perturbation. In case of an electronic angular momentum of 1/2, the energy level shifts in both regimes are analytically described by the so-called Breit-Rabi formula^21^ and corresponding non-linear shifts in the intermediate regime can be calculated; depicted, e.g., in ref.^22^. Such calculations can be used to identify first-order insensitive transitions for specific magnetic fields^19–21^, where differential energy shifts between two states vanish to first-order. Note, clock states at zero magnetic field are used as well^23^, where the absolute energy shift of participating states is zero to first order.

Typically, required magnetic fields are generated by field coils and to ensure stable operation conditions cooling and stable high-power current supplies are required. To further increase fidelities and complexity of quantum applications and/or to enable portable devices, robust and compact experimental setups with highly integrated components are required and being developed^24–28^. Under these circumstances the use of rare-earth magnets to create quantisation fields can be beneficial in contrast to field coils. In the last years, such permanent magnets became more popular for a variety of applications in atomic physics research^29–33^, in particular, due to their high magnetisation and despite their limited tunability of field strengths.

In our manuscript, we introduce a hybrid approach, using an assembly of rare-earth magnets and pairs of field coils, to generate well-controlled quantisation fields with strengths of more than 10 mT. To benchmark the performance of our approach, we use a single trapped Mg^+^ atom as a quantum sensor. Further, we implement a protocol to probe stray magnetic fields with amplitudes of  $\ge 0.2\,$μT oscillating at radio-frequencies enabled by the high stability of our magnetic field setup.

## Experimental Setup

**Figure 1:**
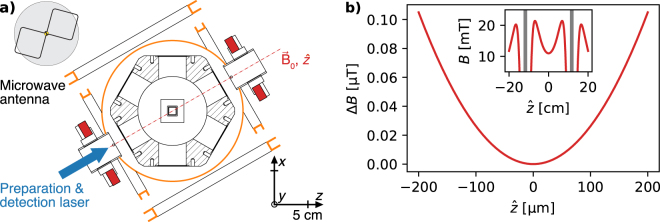
Experimental setup and spatial properties of the solid-state magnet assembly. (**a**) Cross-sectional view of an ultra-high vacuum chamber housing a surface-electrode trap (indicated at the centre), used for spatial manipulation of single atoms. Two sets of rare-earth, ring magnets generate a magnetic (quantisation) field ***B***_0_ along their symmetry axis $\hat{z}$ (indicated by ). In addition, three individual pairs of magnetic field (shim) coils are mounted on corresponding mechanical support structure, marked with (). The shim coils enable fine tuning of ***B***_0_ along longitudinal and orthogonal (vertical and horizontal) directions. Preparation and detection laser beams () enter the chamber along ***B***_0_. A home-built biquad antenna (sketched in the top left corner) is used to apply microwaves around 2*π* × 1,600 MHz for internal state manipulation of the atom. (**b**) The magnetic-field variation of the solid-state magnets close to their geometrical centre (inset shows larger region) along $\hat{z}$, calculated using Eq. 1. From numerical calculations, considering all directions, we infer a diameter of spherical volume ${d}_{{\rm{dsv}}}\simeq 150$ μm, where Δ*B*_0_/*B*_0_ ≤ 1 × 10^−6^.

We equip our experimental setup with a combination of two sets of rare-earth ring magnets and three pairs of field coils (electro magnets) to generate, tune, and stabilise a quantisation field at a strength $|\,{{\bf{B}}}_{0}\,|\,\simeq 10.9$ mT. In Fig. 1a, we sketch the geometry of this hybrid setup. Each set of the solid-state magnets consists of three neodymium (an alloy made of neodymium, iron, and boron) ring magnets that are axially magnetised. Each ring has the following dimensions: 58 mm inner diameter, 102 mm outer diameter, and 4 mm thickness. The vendor specifies the grade of this neodymium in-stock item to be N35, which corresponds to a remanence of ${B}_{r}\simeq 1.17$ T and a temperature coefficient of $\simeq -1.2\,\times {10}^{-3}$ K^−1^ ^34^. We numerically calculate the spatial magnetic field distribution of both sets that are aligned collinear at a distance $d\simeq 223$ mm (distance between facing planes) using the open-source software package RADIA^35,36^. Along their symmetry axis $\hat{z}$, we can also analytically estimate the field distribution. The magnetic-field strength of a single axially magnetised ring is given by^37^:

1 $${B}_{{\rm{ring}}}(\hat{z})=\frac{{B}_{r}}{2}[(\frac{\hat{z}}{\sqrt{{R}_{{\rm{o}}}^{2}+{\hat{z}}^{2}}}-\frac{\hat{z}-D}{\sqrt{{R}_{{\rm{o}}}^{2}+{(\hat{z}-D)}^{2}}})-(\frac{\hat{z}}{\sqrt{{R}_{{\rm{i}}}^{2}+{\hat{z}}^{2}}}-\frac{\hat{z}-D}{\sqrt{{R}_{{\rm{i}}}^{2}+{(\hat{z}-D)}^{2}}})],$$

with inner radius *R*_i_, outer radius *R*_o_, and thickness *D*. We calculate the corresponding field for our magnet assembly by summation of Eq. 1, geometrically offset for each ring. Results of our calculations are shown in Fig. 1b and we, further, numerically estimate the field homogeneity of our magnet configuration in the central region between both sets. Following, we calculate a diameter of spherical volume ${d}_{{\rm{dsv}}}\simeq 150\,$μm, where the relative strength of the magnetic field varies less than 1 × 10^−6^. Note, the specific choice of materials in close proximity to the geometric centre can increase the field inhomogeneity significantly and needs careful consideration in order to estimate the homogeneity within the entire setup. In our setup, we mount each set on a threaded cylinder (one turn equals one millimetre travel) to fine tune *d*. In this way, we can coarsely tune |**B**_0_| by $\simeq 0.11$ mT mm^−1^. For fine tuning of the spatial alignment and the strength, as well as, temporal stabilisation of |**B**_0_|, we deploy the three pairs of field coils (shim coils). All coil pairs can be fed by current-stabilised low-power supplies with a vendor-specified stability of 0.2 × 10^−6^ A and a maximum current of 0.1 A. Two pairs can be used for spatial fine tuning and are aligned transversally to $\hat{z}$: the first pair creates a magnetic field of $\simeq 0.24$ mT for a current of 1 A in the horizontal direction and the second pair tunes the vertical direction with 1.3 mT A^−1^. The third pair of shim coils is aligned along $\hat{z}$ and we can apply a field strength of 0.26 mT A^−1^. In addition, we control the current running in the longitudinal shim coils with our data acquisition system and a resolution of 3 × 10^−6^ A.

Our experimental apparatus for trapping and controlling single atoms is located in a $\simeq$ 100 m^2^ laboratory space that is specified with a temperature stability of better than ±0.3 K. We trap individual ^25^Mg^+^ atoms under ultra-high vacuum conditions with a background gas pressure of below 2 × 10^−9^ Pa in a surface-electrode ion trap. The trap is microfabricated by Sandia National Laboratories and copies of the trap have been previously described^38,39^. A maximum zero-to-peak voltage ${U}_{{\rm{RF}}}\,\simeq \,80$ V oscillating at ${{\rm{\Omega }}}_{{\rm{RF}}}\,\mathrm{/(2}\pi )\simeq 57.3$ MHz is applied to two $\simeq \,2.5$ mm long radio-frequency (RF) electrodes that are 60 μm wide and are separated by $\simeq 210\,$μm. This provides confinement of ions in the *x*-*y* (radial) plane at a distance $h\simeq 83$ μm above the surface. Further, electric (control) potentials are applied to several additional electrodes, in order to confine ions along the *z* (axial) direction. Correspondingly, we find single-ion motional frequencies of $\simeq \,2\pi \,\times 0.8$ MHz (axially) and $\simeq \,2\pi \,\times 2.1$ MHz (radially).

**Figure 2:**
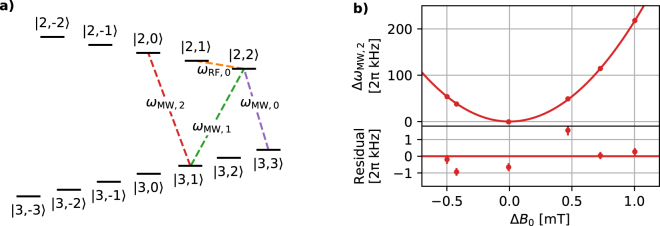
Ground-state hyperfine level scheme and coarse tuning of the quantisation field. (**a**) Relevant Zeeman sub levels of the S_1/2_ hyperfine ground state of ^25^Mg^+^ with a nuclear spin of 5/2 near $|{{\bf{B}}}_{0}|\,\simeq \,10.9$ mT. In our experiments, we use the marked transitions for internal state manipulation via pulsed microwave or radio-frequency radiation, cf. Table 1. (**b**) Measured () and calculated variation Δ*ω*_MW, 2_ of the $|\mathrm{3,1}\rangle \leftrightarrow |\mathrm{2,0}\rangle$ transition frequency ${\omega }_{\text{MW},2}\mathrm{/(2}\pi )\simeq 1762.974\,$ MHz as a function of Δ*B*_0_, and residual (measured - calculated Δ*ω*_MW, 2_) plot. Tuning of the magnetic field strength (on site of a single trapped atom) via variation of the distance between the solid-state magnets within a full span of ${\rm{\Delta }}d\simeq 16\,$ mm. The local magnetic field strength is probed with a relative precision of better than $\simeq \,0.2\times {10}^{-4}$ via the $|\mathrm{3,3}\rangle$ to $|\mathrm{2,2}\rangle$ transition frequency, *ω*_MW, 0_.

The external quantisation field is aligned at an angle of approximately 30° with respect to the *z* axis and lies within the *x*-*z* plane (see Fig. 1a). In Fig. 2a, we illustrate the level scheme of the ^2^S_1/2_ ground state manifold of ^25^Mg^+^ with a nuclear spin of 5/2. Near the field strength ${\boldsymbol{|}}{{\bf{B}}}_{0}|\,\simeq 10.9\,$ mT, the |*F* = 3, *m*_*F*_ = 1〉 to |*F* = 2, *m*_*F*_ = 0〉 hyperfine transition frequency ${\omega }_{\text{MW},2}\mathrm{/(2}\pi )\simeq \mathrm{1,762.974}$ MHz is first-order insensitive to magnetic field changes, while the quadratic frequency deviation is $\simeq 2\pi \times 217$ kHz mT^−2^. Here, *F* denotes the total angular momentum and *m*_*F*_ is the projection of the angular momentum along the magnetic field axis. We keep this notation for labelling purposes only. In case of ${\boldsymbol{|}}{{\bf{B}}}_{0}|\gg 0$, *F* and *m*_*F*_ are inappropriate quantum numbers and, therefore, we calculate level splittings and inter-state coupling strengths numerically. Laser beams (with wavelengths close to 280 nm and *σ*^+^ -polarised) for Doppler cooling to a temperature of $\simeq 1$ mK and state preparation via optical pumping into |3,3〉 of the ^2^S_1/2_ ground state propagate parallel to the magnetic field. For state detection, a single laser beam induces resonant fluorescence and we can discriminate the |3,3〉 (bright) state from the other hyperfine ground (dark) states. Fluorescence photons are detected by a photon-multiplier tube (PMT) detector; more details on our laser setups, state preparation and detection techniques are described in refs.^40–44^. Further, we can coherently manipulate the internal states via a pulsed application of microwaves between ${\omega }_{{\rm{MW}}}\mathrm{/(2}\pi )\simeq \mathrm{1,300}\,$ MHz and $\simeq \mathrm{1,850}\,$ MHz or radio-frequency waves at ${\omega }_{{\rm{RF}}}\mathrm{/(2}\pi )\simeq 55.3\,$ MHz. The microwaves are applied via a home-built biquad antenna^45^ that is geometrically optimised for 2*π* × 1,600 MHz, while the radio-frequency waves are capacitively coupled onto the RF electrodes.

Individual experimental sequences are comprised by about 500 μs of cooling and state preparation, zero to 1.5 s of state manipulations or (near) free evolution, and 100 μs of state detection. Sequences are repeated ${N}_{\exp }\simeq 100$ to 500 times to yield averaged data points (including statistical uncertainties) for fixed parameter settings. More details on raw data analysis in our experiments can be found in ref.^43^. Note, in the following experiments, state preparation can include population transfer from the bright state to any other state of the hyperfine manifold, e.g., |3,1〉 state, via microwave (or radio-frequency) pulses. In turn, state detection, will then include reversed application of pulses to transfer population back into the bright state. After optimisation, we further neglect infidelities of these transfer pulses in the analysis of our experiments; in similar experimental setups infidelities below 10^−4^ have been reported^46^.

## Results

### Tuning and long-term stability of the quantisation field

In dedicated calibration measurements, we tune the orientation and strength of **B**_0_, to enable optimal experimental conditions: We require, firstly, first-order field insensitivity of the |3,1〉–|2,0〉 state splitting and, secondly, optimal state preparation in our experiments. For these calibration experiments, we probe the magnetic field with a single ion via the |3,3〉 to |2,2〉 transition frequency *ω*_MW, 0_, with a field sensitivity of $\simeq \,-2\pi \,\times 21.764$ MHz mT^−1^, cf. Fig. 2 and Table 1. We apply either a single microwave *π* pulse (Rabi sequence, i.e., full population transfer from |3,3〉 to |2,2〉) or two *π*/2 pulses separated by the duration ${T}_{{\rm{Ramsey}}}\le 20$ μs (Ramsey sequence).

**Table 1:** Properties of the four probed hyperfine transitions.

Label	Transition	Trans. frequency	Field sensitivity	Coupling strength	Coherence time
		*ω* [2*π* MHz]	∂*ω*/∂*B* [2*π* MHz mT^−^^1^]	Ω_Coupl._ [2*π* kHz]	*τ* [s]
MW, 0	$|\mathrm{3,3}\rangle \leftrightarrow |\mathrm{2,2}\rangle$	1541.066(4)	−21.764	161(3)	0.42(6) × 10^−3^
MW, 1	$|\mathrm{2,2}\rangle \leftrightarrow |\mathrm{3,1}\rangle$	1655.815(2)	−10.116	38.3(8)	0.9(1) × 10^−3^
MW, 2	$|\mathrm{3,1}\rangle \leftrightarrow |\mathrm{2,0}\rangle$	1762.97381160(1)	±0(1) × 10^−4^ (+0.217 mT^−1^)	28.5(6)	6.6(9)
RF, 0	$|\mathrm{2,2}\rangle \leftrightarrow |\mathrm{2,1}\rangle$	55.260(1)	+5.381	0.28(6)	1.8(2) × 10^−3^

Calculated transition frequencies *ω* and magnetic field sensitivities $\frac{\partial \omega }{\partial B}$ are listed, as well as, a summary of the experimentally applied coupling strengths Ω_Coupl._ and measured coherence times *τ*. All values are taken for a measured magnetic field of |***B***_0_| = 10.9584(2) mT.

A coarse setup of the orientation of **B**_0_, i.e., superposition of the magnetic field with the wave vector of our laser beams for optimal optical pumping into the |3,3〉 state, is ensured by mechanical/geometrical constraints and adjustments of the beam polarisation. Further, we coarsely tune the strength of **B**_0_ by mechanical adjustments of *d*, while monitoring *ω*_MW, 0_ via Rabi sequences. In addition, we record *ω*_MW, 2_ via Rabi sequences to find the field strength corresponding to the first-order field-independent transition, see Fig. 2b.

For fine tuning of **B**_0_, we adjust current amplitudes fed into the shim coils guided by Ramsey sequences probing *ω*_MW, 0_ in multiple iterations: The currents in the vertical and horizontal shim coils are adjusted to minimise |**B**_0_|, i.e., optimising superposition of **B**_0_ with preparation laser beams, while the current in the longitudinal shim coils is optimised for setting |**B**_0_| to its target value within a relative precision of ≤0.1 × 10^−4^. We perform multiple long-term measurements of the passive magnetic-field stability over the course of up to 8 hours with a single ion without re-loading or other systematic variations of experimental parameters. We find maximal variations of the magnetic field strength of $\simeq 0.3\,\times {10}^{-4}$ within five minutes and $\simeq 1.0\,\times {10}^{-4}$ within one hour.

In literature several mechanisms are discussed to influence the stability of fields from permanent magnets and it is distinguished between reversible and irreversible effects. Irreversible effects that lead to a degradation of the magnetisation can be triggered, e.g., by heat, external magnetic fields, and mechanical force. Timescales of this ageing vary strongly with effective amplitudes of these disturbances and are difficult to assess. For example, in our case, a longterm demagnetisation due to the room temperature surrounding may be of about 0.01 within one year, as studied in ref.^47^. On shorter timescales (from minutes to hours), reversible effects due to variations of the surrounding temperature need to be considered. First of all, magnetisation varies proportional to the reversible temperature coefficient and we calculate that in our case (assuming a temperature stability of ±0.3 K) it yields a relative magnetic field stability of better than 3.6 × 10^−4^. Another effect results from thermal expansion of the supporting structure of the magnets with increasing temperature. We estimate this effect to contribute not more than 1.5 × 10^−4^ of field variations. Note, that both of these reversible effects add up in our current setup.

During the following measurement runs, we track magnetic-field strength drifts every five to 20 minutes via variations of *ω*_MW, 0_ within a Ramsey sequence, and readjust current amplitudes of the longitudinal shim coils, accordingly. In this way, we actively stabilise the magnetic field to |***B***_0_| = 10.9584(2) mT.

Finally, we conservatively estimate spatial magnetic field gradients in the vicinity of a single trapped ion from final mechanical setup tolerances and based on the numerical field simulations of the solid-state magnets. We assume that the ion is displaced by less than 2 mm from the geometric centre position $\hat{z}=0$ of the magnet assembly. Therefore, we expect spatial gradients of less than 11 nT μm^−1^ in any direction, neglecting additional contributions, e.g., from contaminating magnetic materials in the trap chip and the surrounding support structures. Note, this corresponds to a spatial variation of *ω*_MW, 2_ of less than 2*π* × 26 μHz μm^−2^.

### Measurements of coherence times

**Figure 3:**
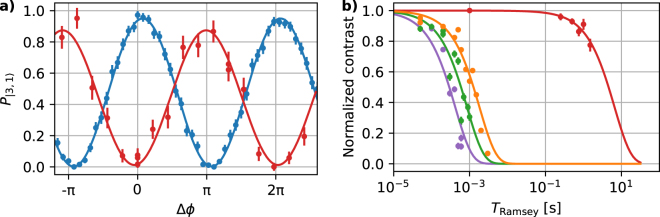
Measurements of superposition-state coherence times via Ramsey spectroscopy. (**a**) As an example, we show the variation of state population *P*_|3,1〉_ as a function of relative (microwave) phase Δ*ϕ* between two *π*/2 pulses, that are separated by *T*_Ramsey_ = 0.001 s () and 1.0 s (); error bars correspond to statistical uncertainties (s.e.m.). Sinusoidal model fits to both data give best values for the achieved contrast of 0.948(7) and 0.86(4), respectively. (**b**) Evolution of contrast normalized to the initial contrast for four different sets of internal state superpositions using states |3,1〉 -|2,0〉 (), |2,1〉 -|2,2〉 (), |3,1〉 -|2,2〉 (), and |3,3〉 -|2,2〉 (), as a function of *T*_Ramsey_; error bars indicate s.e.m.. Individual fits of exponential decays to each data set yield coherence times *τ*, defined as the duration where contrast reaches a level of *e*^−1^. We find corresponding durations of 6.6(0.9) s (), 0.0018(2) s (), 0.0009(1) s (), and 0.00042(6) s () for the four different superposition states, respectively.

In the following, we determine coherence times *τ*–in some literature referred to as the ${{\rm{T}}}_{2}^{\ast }$ relaxation duration–of four different sets of internal state superpositions within the ground state hyperfine manifold, cf. Fig. 2a, in order to further benchmark the performance of our overall setup. In Table 1, we quantify and summarise relevant properties of the probed transitions. We apply the following experimental sequences to measure coherence times: After preparation of the initial state, we create internal state superposition states via a first *π*/2 (microwave or radio-frequency) pulse, wait for fixed durations *T*_Ramsey_, apply a second *π*/2 pulse with variable phase Δ*ϕ* (relative to the phase of the first pulse) and detect the final state. In Fig. 3a, we show, as an example, results of the field-independent superposition states.

We plot the population probability ${P}_{\mathrm{|3,1}\rangle }$ of state $\mathrm{|3,1}\rangle$ as a function of Δ*ϕ* for two different values of *T*_Ramsey_. From sinusoidal model fits to the data, we determine the contrast of such Ramsey sequences for all four sets of superposition states for variable *T*_Ramsey_ and show these results in Fig. 3b. In a final analysis step, we determine *τ*, i.e., the duration *T*_Ramsey_ after which the initial contrast decayed to *e*^−1^, by exponential model fits to each data set. We find a coherence time of 6.6(9) s for the field-independent superposition states, while coherence times are shorter than two milliseconds for all other superposition states; all results are summarised in Table 1. Measured decoherence rates Γ = 2*πτ*^−1^ increase linearly as a function of the corresponding magnetic-field sensitivities and suggesting significant magnetic-field fluctuations on time scales between a few hundred microseconds and a few seconds. From additional experiments with less stable power supplies feeding the shim coils, we estimate that noise levels from the relevant current supplies contribute less than 2*π* × 0.002 Hz to the lowest decoherence rates of 2*π* × 0.15(3) Hz (for the field insensitive transition). Further, we assume that the limited thermal stability of the permanent magnets contributes significantly to field noise in our setup. A temperature variation of about 45 mK on timescales faster than the bandwidth ($\simeq 1$ mHz) of our active stabilisation translates to a field variation of $\simeq 0.8\,$ µT and would suffice to explain the observed decoherence rates. For reference, we infer the amplitude of background/stray magnetic field noise to be ≤0.1 μT from another experimental setup in our laboratory^48^. Note, we ensure that leakage from our laser beams contribute less than 2*π* × 0.08 Hz (for all probed transitions).

### Sensing of oscillating magnetic fields

**Figure 4:**
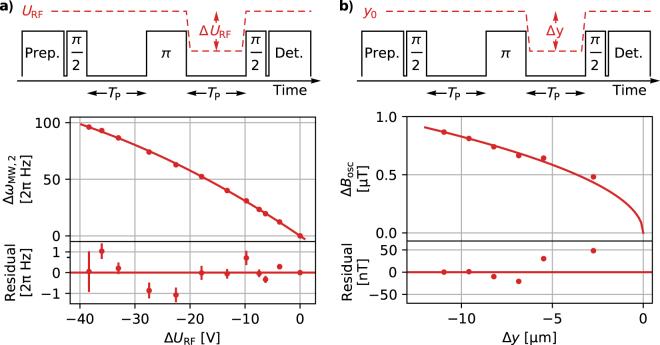
Sensing of oscillating magnetic fields originating from stray radio-frequency currents. Detection of variations of field strength *B*_osc_ via spin-echo sequences that record phase accumulation from induced differential (frequency dependent) a.c. Zeeman shifts, while the ion is in a superposition state of $|\mathrm{3,1}\rangle$ and $|\mathrm{2,0}\rangle$. For fields oscillating at Ω_osc_ = Ω_RF_ = 2*π* × 57.3 MHz and our setup, we calculate a quadratic sensitivity of 2*π* × 4.783 Hz μT^−2^. Pulse sequences of the spin-echo experiments varying Δ*U*_RF_ and the ion position along *y* respectively, times not to scale. (**a**) Differential a.c. Zeeman shifts Δ*ω*_MW, 2_ as a function of Δ*U*_RF_, i.e., systematic variation of stray currents ∝ *U*_RF_. Uncertainties of data points are smaller than the markers. We find a slope of 2*π* × 20.77(7)mHzV^−2^ from a model fit and conclude *B*_osc_ = 5.239(8) μT for our typical operation condition *U*_RF_ = 79.5 V. (**b**) Spatial variation of *B*_osc_ along the *y* direction. Data is in agreement with a model fit with a slope of $\delta {B}_{osc}/\sqrt{(\delta y)}$ = 0.262(3) μT μm^−1/2^.

In a first application, we use the clock transition for sensing of oscillating magnetic fields **B**_osc_ that originate from stray currents with unknown amplitude (∝ *U*_RF_) in the two RF electrodes. We consider that these fields predominantly lie in the *x*-*y* plane, due to the symmetry of the electrode structure. Under this assumption and from basic atomic properties, we calculate the frequency dependent a.c. Zeeman shift^49^ of the probed transition, and find a quadratic sensitivity of 2*π* × 4.783 Hz μT^−2^ to fields oscillating at Ω_RF_. To further characterise **B**_osc_, we apply the following experimental (spin-echo) sequence and detect phase accumulations from differential a.c. Zeeman shifts due to a variation of *U*_RF_: After preparation of |3,1〉, a *π*/2 pulse to create a |3,1〉−|2,0〉 superposition, and a free evolution duration *T*_P_, we apply a *π* pulse in phase with the previous pulse. After an additional duration *T*_P_, during which we ramp down and back the radio-frequency voltage by Δ*U*_RF_ within ramp durations of ≤80 μs, we conclude the experimental sequence with a second *π*/2 pulse (again, in phase with the previous pulses) and detection of the |3,1〉 state, cf. Fig. 4a. Note, the spin echo sequence makes results insensitive to quantisation-field fluctuations slower than the time scale of an individual sequence ($\simeq 1$ s). In subsequent measurements, we vary *T*_P_ to up to 1.2 s for fixed Δ*U*_RF_ to determine the phase accumulation from differential a.c. Zeeman shifts. Corresponding results as a function of Δ*U*_RF_ are shown in Fig. 4a. A quadratic model fit to this data yields a slope of 2*π* × 20.77(7) mHzV^−2^ and from this we infer an oscillating magnetic field strength of *B*_osc_ = 5.239(8) μT for *U*_RF_ = 79.5 V.

Next, we measure the spatial dependence of this field along the *y* axis. We deploy a similar spin-echo sequence as described above, but vary the position Δ*y* of the ion, respectively to its initial position *y*_0_, within the second free evolution duration for fixed *U*_RF_, cf. Fig. 4b. In such sequences, the ion position is varied by applying electric control fields. We calibrate relative ion displacements in dedicated measurements to within ±0.2 μm and ensure that displacements in all other directions are less than $\simeq \,1.0$ μm for maximal *y* displacements. We observe a linear variation of Δ*ω*_MW, 2_ as a function of Δ*y* with a slope of 2*π* × 327(7) mHz μm^−1^ and attribute this to differential a.c. Zeeman shifts. Note, to explain the observed frequency shift by a spatial variation of static magnetic fields only, it would require local gradients of $\simeq 1.2\,$ mT μm^−1/2^. In comparison, we refer to our estimation of global linear gradients of less than 11 nT μm^−1^ (see above) and judge the presence of such large (static) local gradients to be unlikely in our setup. Consequently, we show in Fig. 4b the variation of *B*_osc_ with a non-linear slope of $\delta {B}_{{\rm{osc}}}/\sqrt{\delta y}=\mathrm{0.261(3)}\,$ μT μm^−1/2^.

## Discussion

We describe a hybrid approach for generating stable magnetic fields, with a field strength around 10.9 mT and a calculated spatial variation of less than 10^−6^ within a diameter of spherical volume of 150 μm, using a combination of rare-earth magnets and magnetic field coils powered by stable low-power current supplies. We coarsely tune the magnetic field by mechanical adjustments of the permanent magnets and use the field coils for fine tuning. In our experiments, we use a single trapped Mg^+^ atom to probe the field characteristics. We find a passive long-term temporal stability of $\simeq 1\,\times {10}^{-4}$ over the course of one hour. In addition, we implement a feed-back loop for active field stabilisation with a bandwidth of about 1 mHz to better than 2 × 10^−5^ via re-adjustments of currents in the field coils. Further, we benchmark the short-term performance of our setup by measurements of coherences of internal state superpositions and find coherence times of up to 6.6(9) s. We assume that the short-term stability is limited by the passive (thermal) stability of the permanent magnets and the bandwidth of the active field stabilisation. In a first quantum sensing application, we probe magnetic fields oscillating at 2*π* × 60 MHz that originate from currents running in our trapping structure. We measure the magnitude with a quadratic sensitivity of 2*π* × 4.783 Hz mT^−2^ and spatial variation within about ten micrometers. In an extension of our measurements, complete, i.e., local amplitude and phase information of the oscillating field can be recorded^50^. Numerical simulations of the oscillating magnetic fields can be compared to our results and, in turn, would yield detailed understanding of electronic properties of trapping structures that are used for quantum simulation^42^ and related fields of research.

The stability of our hybrid approach can be further increased in several ways. First of all, we can increase the bandwidth and accuracy of the active stabilisation. In addition, we can combine permanent magnets with different reversible temperature coefficients in arrays that create magnetic fields that are intrinsically robust against thermal variations^51^. Another way can be to use a support structure that is engineered to counteract on the change of magnetisation due to thermal drifts by a change in the distance between the magnet sets. The magnetic field noise floor can be improved via implementation of shielding against stray magnetic fields as, e.g., demonstrated in ref.^26^. Finally, adapted and optimised geometries of the solid-state magnets can yield smaller footprints, while increasing regions of homogeneous field distribution, and variable field strengths. We conclude that improved versions our hybrid approach are of particular importance for setups that have strict power and load requirements, while being cost effective. Thus, it enables more compact and robust developments in a variety of applications.

### Data availability

The datasets generated and analysed during the current study are available from the corresponding author on reasonable request.
